# Unnatural-cause mortality patterns of Northern Finnish men and women diverge in adolescence – A 52-year follow-up

**DOI:** 10.1016/j.pmedr.2021.101337

**Published:** 2021-02-23

**Authors:** Juho-Antti Junno, Lasse Pakanen, Petteri Oura

**Affiliations:** a Cancer and Translational Medicine Research Unit, Faculty of Medicine, University of Oulu, Oulu, Finland; b Department of Archaeology, Faculty of Humanities, University of Oulu, Oulu, Finland; c Archaeology, Faculty of Arts, University of Helsinki, Helsinki, Finland; d Forensic Medicine Unit, Finnish Institute for Health and Welfare, Oulu, Finland; e Department of Forensic Medicine, Research Unit of Internal Medicine, Medical Research Center Oulu, University of Oulu, Oulu, Finland; f Department of Forensic Medicine, University of Helsinki, Helsinki, Finland; g Forensic Medicine Unit, Finnish Institute for Health and Welfare, Helsinki, Finland; h Center for Life Course Health Research, Faculty of Medicine, University of Oulu, Oulu, Finland

**Keywords:** Mortality, Death, Forensic medicine, Cohort studies, Northern Finland Birth Cohort 1966, Epidemiology

## Abstract

The Finnish population has a long life expectancy but ranks high in unnatural deaths on the European scale. Mortality has historical regional discrepancy in Finland, as Northern Finns are overrepresented in both natural and unnatural deaths. This study aimed to characterize the age- and sex-related trends in unnatural mortality among Northern Finns. Altogether 12 143 individuals, constituting >95% of births in Northern Finland in 1966, were followed up for a median of 52 years. The mortality patterns of this population were studied using death record data. Crude annual mortality rates were calculated and graphed for 10-year age strata (all-cause, natural-cause, and unnatural-cause mortality, as well as accident, suicide, and homicide mortality). Cox regression was used to analyze the sex discrepancy in mortality. A total of 874 deaths (7.2%) occurred during the follow-up period. Women had 47% and 73% lower risks of any death and unnatural death than men, respectively. From the second decade of life onwards, the unnatural mortality of men was 3–5 times that of women. Accident and suicide mortality rates of men were 2–13 and 2–3 times those of women, respectively. Homicides were rare among either sex. We conclude that Northern Finnish women have a substantially lower risk of all-cause mortality and unnatural mortality than men. To aid the development of preventive strategies, future studies should aim to identify the underlying factors behind unnatural mortality. Primarily, emphasis should be placed on the increased mortality of men from the second decade of life onwards.

## Highlights

• Northern Finnish women had lower risks of any death and unnatural death than men.
• After early life, the unnatural mortality of men was 3–5 times that of women.
• Accident and suicide mortality rates of men were ≥ 2 times those of women.
• Homicides were rare among either sex.

## 1 Introduction

Death, being the event to mark the irreversible end of a person’s life, serves an important function in public health surveillance (Centers for Disease Control and Prevention (CDC), 2006, Mathers et al., 2005, Brooks and Reed, 2015, Gill, 2017, Aung et al., 2010). Systematic collection, analysis and dissemination of data related to mortality and cause of death enable the monitoring of trends in society and inform decision-making in public health (Centers for Disease Control and Prevention (CDC), 2006, Mathers et al., 2005). Mortality statistics are generated according to the causes of death reported on death certificates (Aung et al., 2010). As the cause of death defines the disease or injury primarily responsible for the fatality, it constitutes an obvious target in the prevention of similar deaths in the future (Brooks and Reed, 2015).

In deaths caused by a disease, the manner of death is natural, whereas deaths due to external causes (such as accidents, adverse healthcare events, suicides, and homicides) are classified as unnatural (Gill, 2017). In rare cases, the cause or manner of death may remain undetermined (Gill, 2017). From the public health perspective, statistics related to unnatural deaths are of particular importance, as these deaths are relatively common (GBD 2017 Causes of Death Collaborators, 2018) and mostly preventable. The substantial financial costs associated with premature unnatural deaths underline the need for their effective prevention (Rice et al., 1985, Corso et al., 2007, Florence et al., 2015).

Mortality patterns and cause-of-death distributions vary across the world (GBD 2017 Causes of Death Collaborators, 2018). Recently, reports of mortality with a special focus on unnatural causes have been published in several populations (Aparici and Byard, 2019, Panda and Mishra, 2020, Lukaschek et al., 2012, Ossei et al., 2019, Islam and Islam, 2003) and subgroups (e.g., children and adolescents (Athani et al., 2017, Holakouie-Naieni et al., 2016, Johansson et al., 2005), women (Kumar et al., 2013, Ali et al., 2003, Mahesan Paul et al., 2016), the elderly (Vadysinghe et al., 2018, Kumar and Verma, 2014), the homeless (Slockers et al., 2018), individuals with psychiatric conditions (Swaraj et al., 2019, Walker et al., 2015, Bachmann, 2018) and somatic conditions (Gorton et al., 2018, Webb et al., 2014, Wang et al., 2015, Heiberg Brix et al., 2019)). In Finland, the death certification process is reliable (Mathers et al., 2005) and mortality rates are relatively low (GBD 2017 Causes of Death Collaborators, 2018), age-standardized all-cause mortality being around 1200 and 800 per 100 000 per year for men and women, respectively (Statistics Finland, 2019a). However, over 50% of the deaths of Finnish adolescents and young adults are due to unnatural causes, mostly accidents and suicides (Statistics Finland, 2019a). Compared to other European countries, Finland ranks undesirably high in accident and suicide mortality (Somerkoski et al., 2014, Holopainen et al., 2014).

Within the Finnish population, regional disparity in health outcomes has prevailed since at least the 1930s (Pitkänen et al., 2000, Koskinen, 1995). It has been repeatedly observed that individuals born in Northern Finland have higher morbidity and mortality than individuals born in the south or west of the country. Despite the recent overall development in life expectancy, geographical discrepancies in natural and unnatural mortality persist (Musakka, 2008, Huttunen, 2018, Lantto, 2015, Somerkoski et al., 2014, Partonen et al., 2020), warranting further investigations of the mortality patterns among Northern Finns in particular. In the present study, we had the opportunity to further address the regional differences in mortality by characterizing the deaths of Northern Finns. With our data, we aimed to strengthen the basis for future studies to identify the explanatory factors underlying this regional discrepancy, and finally, to prevent premature unnatural deaths.

This birth cohort study aimed to characterize age- and sex-related trends in unnatural mortality among Northern Finns born in 1966. Altogether 12 143 coeval individuals, originally constituting >95% of the births in Northern Finland in 1966, were followed up until midlife for a median time of 52 years. Using the Finnish death record data, the mortality patterns of this population were studied from birth until the sixth decade of life.

## 2 Materials and methods

### 2.1 Study protocol

This study is based on the Northern Finland Birth Cohort 1966 (NFBC1966) dataset (University of Oulu, 2020). The NFBC1966 is an unselected, population-based birth cohort, initiated in Northern Finland (i.e., the provinces of Oulu and Lapland) in 1965, when pregnant mothers with expected delivery dates within the calendar year 1966 were asked to participate in prospective data collections (Rantakallio, 1988). Of all the 12 527 births that occurred in Northern Finland at the time, 12 231 (including stillbirths and live births) were recorded in the NFBC1966 (Northern Finland Cohorts, 2020), indicating a 97.6% coverage of the source population.

For this study, we linked the NFBC1966 database with the official death records from Statistics Finland, using the permanent unique personal identification codes issued to all Finnish citizens. The follow-up period was from 1965 until the end of 2018, i.e., from the prenatal period until the age of 52 (range 51–53 years). As 88 individuals had declined or their data were otherwise unavailable, our analysis was based on 12 143 individuals.

Approval for the NFBC1966 study and the use of associated death records was obtained from the Ethics Committee of the Northern Ostrobothnia Hospital District and from Statistics Finland. The internal Scientific Committee of NFBC1966 also approved the research plan of this study. The study met the institution’s guidelines for protection of human subjects concerning their safety and privacy. The researchers accessed all the data in pseudonymized format, with personal identification codes removed. Informed consent was obtained from all the participants and/or their guardians.

### 2.2 Death records

In all deaths, a cause-of-death investigation is mandatory by law in Finland (the Act relating to cause-of-death investigation 459/1973). The determination of cause of death is carried out either by means of a medical examination with or without a clinical autopsy, or a police-led forensic examination which often involves a medico-legal autopsy (Kuvaja et al., 2018). Medical examination typically suffices in cases in which a natural cause of death seems obvious, constituting approximately 80% of deaths. In contrast, a police-led investigation is required in all cases that involve suspicion of homicide, suicide, accidental death, a medical or surgical adverse event, or an occupational disease, and when the death is sudden or unexpected. Conventionally, the autopsy rate in Finland is high (Saukko, 1995, Lunetta et al., 2007).

The outcome of the cause of death investigation is communicated via a death certificate (National Institute for Health and Welfare (THL), 2020). The certificate states (among other information) the date of death and the primary cause of death with contributing causes according to the International Classification of Diseases (ICD). The manner of death (disease, occupational disease, accident, medical or surgical adverse event, suicide, homicide, war, or undetermined) is selected according to the primary cause and the circumstances of death. Once completed, all death certificates are sent to forensic pathologists at the Finnish Institute for Health and Welfare (Terveyden ja hyvinvoinnin laitos; a public health expert agency operating under the Ministry of Social Affairs and Health) for an independent review. Incorrectly completed or flawed certificates are returned for necessary corrections or further actions, and accepted certificates are forwarded to Statistics Finland (Tilastokeskus).

Statistics Finland is a long-standing public authority, founded in 1865, which produces the majority of Finnish official statistics. Statistics Finland also maintains comprehensive death certificate archives and produces cause of death statistics (Statistics Finland, 2020). A validation study of the Finnish death certification system concluded that the death certificate form, certification practices and cause of death validation serve the coding of causes of death appropriately (Lahti and Penttilä, 2001).

For this study, we used the dates, primary causes, and manners of death accumulated from 1965 until the most recent update of the Statistics Finland database, i.e., the end of 2018. As the deaths of Finnish residents abroad are generally notified to the Finnish authorities (Ministry for Foreign Affairs, 2021), individuals with no death records in the Statistics Finland database were assumed to be alive. Time of death was obtained to an accuracy of one month (for example January 1966), which was considered acceptable to fulfill the aims of this study. In order to track the age-related patterns of mortality in informative, yet reasonably sized strata, deaths were stratified according to age at death at regular intervals, namely 0–9, 10–19, 20–29, 30–39, 40–49, and 50+ years.

Over time, various versions of the ICD coding system have been used (ICD-7 in 1965–1968, ICD-8 in 1969–1986, ICD-9 in 1987–1995, and ICD-10 in 1996–2018). Cases with disease codes as the primary cause of death were classified as ‘natural’ deaths, and cases with external cause codes were classified as ‘suicide’, ‘homicide’ and ‘accident’, in congruence with the original archived data; these three classes were referred to as ‘unnatural’ deaths. Individuals with an undetermined manner of death or unknown primary cause of death were classified as ‘undetermined’.

### 2.3 Statistical analysis

IBM SPSS Statistics software version 25 (Armonk, NY, USA) was used to perform the data analysis and generate Kaplan-Meier curves for mortality over the follow-up. Microsoft Excel software version 2005 (Redmond, WA, USA) was used to create mortality plots and pie charts. The threshold for statistical significance was set at P = 0.05.

Sex difference in all-cause, natural-cause, and unnatural-cause mortality over the follow-up was analyzed using Cox regression. In the natural and unnatural-cause models, outcome events were defined respectively, and individuals were censored at the occurrence of a non-outcome death or at the end of the follow-up. Men were chosen as the reference group to which women were compared, and hazard ratios (HRs) with their 95% confidence intervals (CIs) were extracted from the regression output. As clear sex differences were observed, the subsequent analyses were stratified by sex.

Crude estimates for the annual mortality rate in each age stratum (given per 100 000 individuals per year) were calculated as (D/L)/P × 100 000, where D = number of applicable deaths recorded during the period (for all-cause mortality, all deaths were applicable; for unnatural-cause mortality, only unnatural deaths were applicable; etc.), L = length of the period in years, and P = mean population size during the period (Centers for Disease Control and Prevention (CDC), 2006). The estimates of each age stratum were then plotted in order to illustrate the age-related trends in mortality across the follow-up.

## 3 Results

**Fig. 1:**
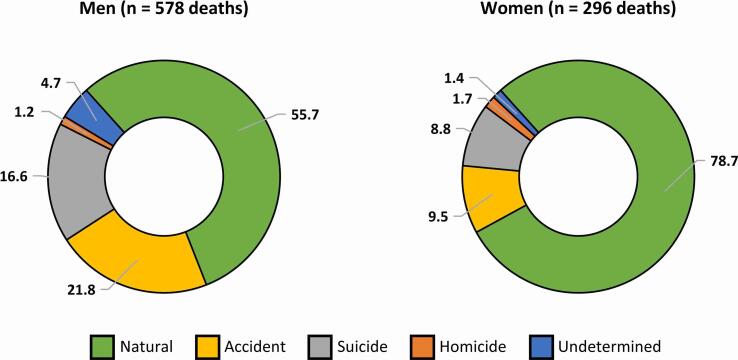
Breakdown of deaths accumulated in the sample over the follow-up. Numbers are percentages.

The study population comprised 12 143 individuals, of whom 51.2% were men (n = 6219) and 48.8% were women (n = 5924), who were followed up for a median of 52 years. Altogether 874 deaths occurred during the follow-up period, with the cumulative mortality rate of the population totaling 7.2% until the end of 2018. Of all deaths, 63.5% were natural (n = 555), 33.0% were unnatural (n = 288), and 3.5% were undetermined (n = 31). A breakdown of these is visualized in Fig. 1.

**Fig. 2:**
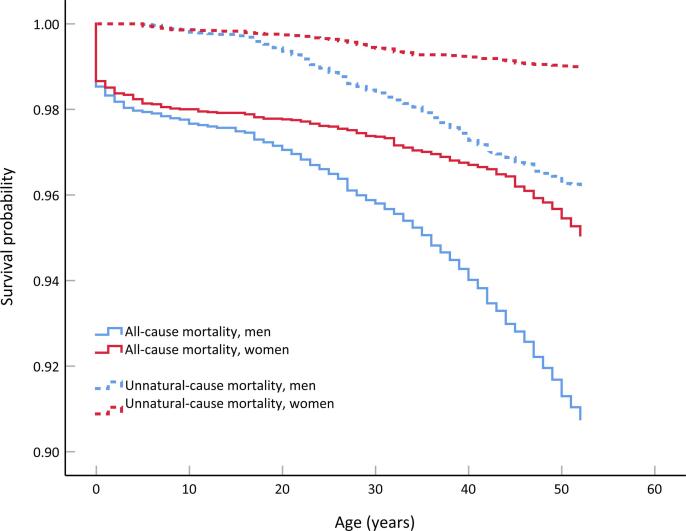
Kaplan-Meier curves illustrating all-cause and unnatural-cause mortality among men and women.

Fig. 2 presents the Kaplan-Meier survival curves for men and women, and Table 1 shows the corresponding hazard ratios from Cox regression. In comparison to men, women had a 47% lower risk of death over the follow-up period. Sub-analysis by manner of death showed the risks for natural and unnatural death to be 25% and 73% lower among women, respectively.

**Table 1:** Hazard ratios for sex difference in all-cause, natural-cause, and unnatural-cause mortality over the 52-year follow-up.

Event	Men		Women		
	Events	HR	Events	HR	(95% CI)
Death due to any cause	578 (9.3%)	1 (ref)	296 (5.0%)	0.53	(0.46–0.61)
Death due to natural cause	322 (5.2%)	1 (ref)	233 (3.9%)	0.75	(0.63–0.88)
Death due to unnatural cause	229 (3.7%)	1 (ref)	59 (1.0%)	0.27	(0.20–0.35)

CI = Confidence interval, HR = Hazard ratio, Ref = Reference group in Cox regression analysis.

**Fig. 3:**
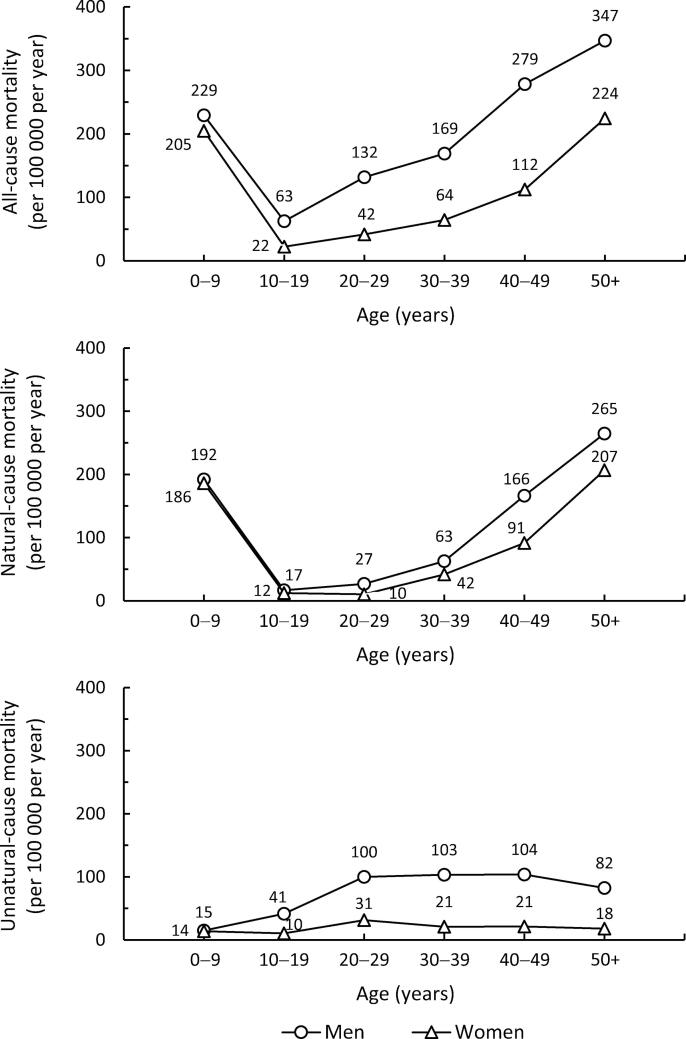
Crude rates for annual all-cause, natural-cause, and unnatural-cause mortality in age strata among men and women.

Fig. 3 shows the crude mortality rates for all-cause, natural-cause, and unnatural-cause mortality across age strata among each sex. All-cause mortality followed a similar pattern among both sexes, namely a steep drop after early life and a gradual rise thereafter, though men had somewhat higher mortality than women in all age strata (63–347 vs 22–224 per 100 000 per year). Although natural-cause mortality was somewhat higher among men throughout the follow-up, it explained less than half of the sex discrepancy in all-cause mortality.

In unnatural-cause mortality, the patterns of men and women clearly diverged in adolescence. Whereas women maintained a relatively stable unnatural-cause mortality rate throughout the follow-up (10–31 per 100 000 per year), the corresponding mortality rate of men climbed to the level of around 100 per 100 000 per year. From the second decade of life onwards, the mortality rate of men was 3–5 times that of women, with unnatural-cause mortality explaining at least half of the sex discrepancy in all-cause mortality.

**Fig. 4:**
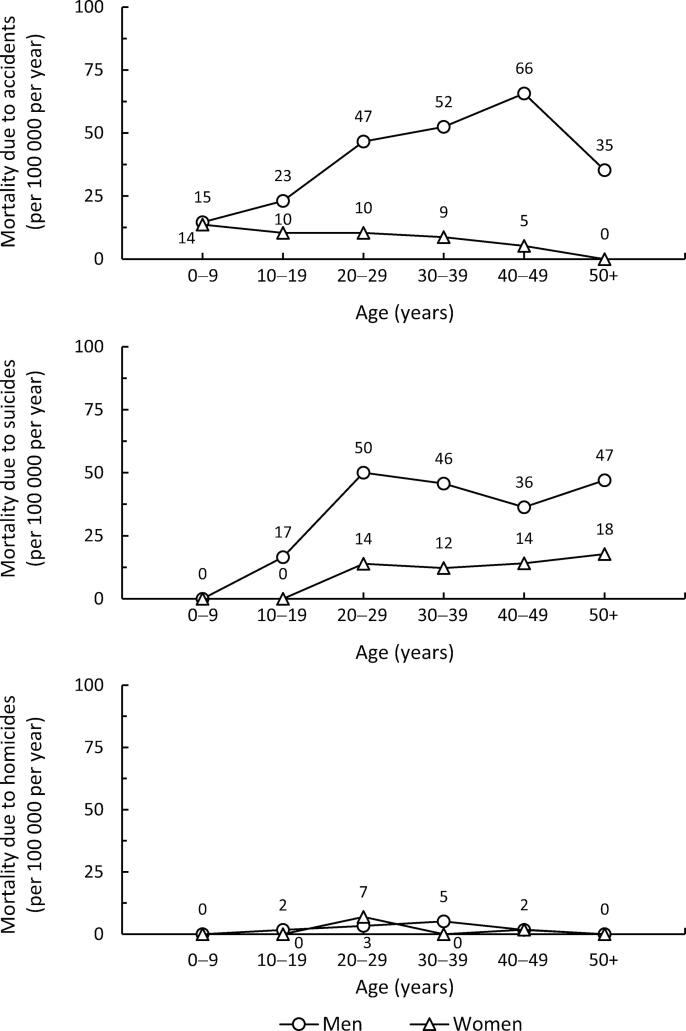
Crude rates for annual mortality due to accidents, suicides, and homicides in age strata among men and women.

Fig. 4 provides further breakdown of unnatural deaths, demonstrating mortality due to accidents, suicides and homicides across age strata. As regards mortality due to accidents, the patterns of men and women were distinctly different. Women had consistently low accident mortality from early life until the end of the follow-up, whereas men showed an increasing trend by age. After early life, accident mortality among men was 2–13 times that of women. In suicide mortality, men showed an increasing trend until the third decade of life, with a relatively stable pattern thereafter. Similarly, women had a mild increase between the second and third decades of life, with a stable pattern thereafter. After early life, suicide mortality among men was 2–3 times that among women. Homicides were a rarity among both sexes.

## 4 Discussion

Although the Finnish population has relatively long life expectancy and low all-cause mortality (GBD 2017 Causes of Death Collaborators, 2018), it ranks undesirably high in unnatural deaths on the European scale (Somerkoski et al., 2014, Holopainen et al., 2014). Moreover, it has historical regional discrepancies in mortality (Pitkänen et al., 2000, Koskinen, 1995), with Northern Finns overrepresented in both natural and unnatural deaths. This birth cohort study of 12 143 individuals with a median follow-up of 52 years aimed to characterize age- and sex-related trends in unnatural mortality among Northern Finns. During the follow-up, a total of 874 deaths accumulated in the sample. Compared to men, women had half the risk of all-cause mortality and a three-quarters lower risk of unnatural death. From the second decade of life onwards, the unnatural-cause mortality of men was 3–5 times that of women and explained over half of the sex discrepancy in all-cause mortality. Accident and suicide mortality were higher among men than women in all age strata after early life.

In our data, accidents and suicides were clearly the most typical causes of unnatural death, accidents being slightly more common among both sexes. Homicides were rare among either sex. Among women, there were fewer deaths overall and a higher natural-to-unnatural ratio than among men. Temporal variation in the number of suicides in Finland has been clear, especially among men, with numbers peaking in the late 80s and decreasing afterwards (Statistics Finland, 2019b). However, we did not detect a similar trend in our study population as suicide mortality remained relatively stable from the second decade of life onwards. Considering that economic recessions are associated with increased suicide rates (Reeves et al., 2015), it is surprising that the effects of the early 90s economic depression on suicide mortality seem to be minor in our dataset, even though the NFBC1966 members were at a high-risk age (i.e., around 24-year-olds) at the time.

Interestingly, the unnatural mortality patterns of men and women clearly diverged during the second decade of life, and this discrepancy lasted until the end of the follow-up. It thus seems that the underlying factors contributing to sex discrepancy are already present, or at least come into effect, in adolescence. From the aspect of prevention, the second decade of life appears to be an important period for interventions, especially regarding the men of this population. Another peculiar trend in our data was the plateau in unnatural mortality among men from the third to the fifth decade of life, after which unnatural mortality and accidental deaths in particular appeared to drop slightly. It will be interesting to see whether this decrease becomes more apparent over time. If it did, this would suggest that the age of 20–49 is a period of increased susceptibility to unnatural death among the men of this population. In future studies, it would be highly relevant to identify the underlying factors in order to understand and prevent deaths that occur during the susceptibility periods.

Previous reports have described varying proportions of accidents, suicides and homicides in Finnish and foreign populations. In the domestic context, Northern Finns seem to a have higher proportion of accident deaths (17% vs 10%) and suicide deaths (14% vs 8%) as well as a more prominent male-to-female ratio (4.5 vs 3.9 for accidents, 3.7 vs 3.0 for suicides) in comparison to all working-age Finns (Statistics Finland, 2019a). Globally, around 14% of all deaths are reported as unnatural (17% for men, 10% for women) (GBD 2017 Causes of Death Collaborators, 2018). The fact that these numbers are manifold in the present dataset (40% for men, 20% for women) is primarily explained by the younger population. Roughly, similar overall trends in sex discrepancy (i.e., male overrepresentation) and age-related mortality (i.e., increase from the second decade of life onwards) can be observed from both datasets. While detailed comparisons to external datasets are challenging and potentially biased due to differing study designs, we speculate that a multifactorial body of demographic, cultural, genetic and societal differences explain the differences in mortality between populations on a global level as well as between Finnish subpopulations. Moreover, the accuracy and reliability of the cause-of-death investigation (in terms of, e.g., autopsy rate) is another likely entity explaining the differences between datasets.

The strengths of this study stem from its unselected sample and reliable death records. The sample size was relatively large, consisting of a Northern Finnish 1966 generation of >12 000 individuals followed up for a median of 52 years. The data collected from the follow-up period allowed us to reflect on mortality in early life, early adulthood, and midlife, enabling the analysis of longitudinal age-related trends of mortality among this population. The unselected, population-based nature and high coverage of the cohort (>95% of applicable births) reduce the risk of selection bias and enhance the applicability of the findings. The mortality data and causes of death were derived from the comprehensive Finnish cause-of-death database, which operates in a systematic manner and has been concluded to be sufficiently reliable, with double-checking of certificates before archiving.

This study has several limitations. Although the sample provided an in-depth profile of the mortality patterns of Northern Finns included in the birth cohort of 1966, we had no data on individuals outside this cohort. As the individuals were followed up from birth until their current median age of 52 years, we unfortunately had no data to investigate mortality and causes of death in later life. These, as well as the potential underlying factors behind mortality patterns, will hopefully be addressed in future studies. In spite of the large, almost maximally comprehensive sample of the source population, there were few accumulated deaths within some of the age strata, which should be taken into account when interpreting the mortality statistics provided here. This was also the reason why further statistics regarding individual causes of death could not be produced here.

## 5 Conclusion

This birth cohort study of 12 143 Northern Finns found clear age- and sex-related trends in unnatural mortality during the follow-up. Women had a substantially lower risk of both all-cause mortality and unnatural mortality than men. From the second decade of life onwards, the unnatural-cause mortality of men was 3–5 times that of women, and explained over half of the sex discrepancy in all-cause mortality. Further breakdown of the data showed that accident and suicide mortality were higher among men than women in all age strata after early life. In order to aid the development of interventions and preventive strategies, future studies should aim to identify the underlying factors behind these unnatural deaths. Primarily, emphasis should be placed on the increased mortality of men from the second decade of life onwards. In the future, it will be interesting to see the development of the mortality patterns of this population in old age.

## Funding

The NFBC1966 study received financial support from

• University of Oulu, Finland (Grant no. 65354, 24000692)
• Oulu University Hospital, Finland (Grant no. 2/97, 8/97, 24301140)
• Ministry of Health and Social Affairs, Finland (Grant no. 23/251/97, 160/97, 190/97)
• National Institute for Health and Welfare, Helsinki, Finland (Grant no. 54121)
• Regional Institute of Occupational Health, Oulu, Finland (Grant no. 50621, 54231)
• European Regional Development Fund ERDF (Grant no. 539/2010 A31592)

## Compliance with ethical standards

Ethics approval: Approval for the NFBC1966 study and the use of associated death records was obtained from the Ethics Committee of the Northern Ostrobothnia Hospital District and from Statistics Finland. The internal Scientific Committee of NFBC1966 also approved the research plan of this study. All procedures were in accordance with the ethical standards of the institutional and/or national research committee and with the 1964 Helsinki declaration and its later amendments or comparable ethical standards.

Consent to participate: Informed consent to participate in a scientific study and publish the related results was obtained from all individual participants (and their guardians) included in the study, and all procedures were in accordance with the ethical standards of the institutional and/or national research committee and with the 1964 Helsinki declaration and its later amendments or comparable ethical standards.

Consent for publication: Informed consent to participate in a scientific study and publish the related results was obtained from all individual participants (and their guardians) included in the study, and all procedures were in accordance with the ethical standards of the institutional and/or national research committee and with the 1964 Helsinki declaration and its later amendments or comparable ethical standards.

Availability of data and material: The datasets generated and analysed during the study are not made publicly available. The NFBC1966 data is available from the University of Oulu, Infrastructure for Population Studies. Permission to use the data can be applied for research purposes via electronic material request portal. In the use of data, we follow the EU general data protection regulation (679/2016) and Finnish Data Protection Act. The use of personal data is based on cohort participant’s written informed consent at his/her latest follow-up study, which may cause limitations to its use. Please, contact NFBC Project Center and visit the cohort website (www.oulu.fi/nfbc) for more information.

## CRediT authorship contribution statement

**Juho-Antti Junno:** Conceptualization, Methodology, Writing - original draft. **Lasse Pakanen:** Conceptualization, Methodology, Writing - review & editing. **Petteri Oura:** Conceptualization, Methodology, Formal analysis, Data curation, Writing - original draft, Supervision.

## Declaration of Competing Interest

The authors declare that they have no known competing financial interests or personal relationships that could have appeared to influence the work reported in this paper.
